# Metabolomic and Transcriptomic Profiling Provide Novel Insights into Fruit Ripening and Ripening Disorder Caused by 1-MCP Treatments in Papaya

**DOI:** 10.3390/ijms22020916

**Published:** 2021-01-18

**Authors:** Senlin Zheng, Yanwei Hao, Silin Fan, Jiahui Cai, Weixin Chen, Xueping Li, Xiaoyang Zhu

**Affiliations:** Guangdong Provincial Key Laboratory of Postharvest Science of Fruits and Vegetables/Engineering Research Center for Postharvest Technology of Horticultural Crops in South China, Ministry of Education, College of Horticulture, South China Agricultural University, Guangzhou 510642, Guangdong, China; zsl1966028969@126.com (S.Z.); yanweihao@scau.edu.cn (Y.H.); 18733508721@139.com (S.F.); Jh.Cai_chn@outlook.com (J.C.); wxchen@scau.edu.cn (W.C.); lxp88@scau.edu.cn (X.L.)

**Keywords:** metabolome, transcriptome analysis, 1-methylcyclopropylene, ripening disorder, phenylpropane pathway, fruit quality, papaya

## Abstract

Treatment with 1-methylcyclopropylene (1-MCP) is an effective technique to preserve fruits, but inappropriate treatment with 1-MCP causes a ripening disorder (rubbery texture) in papaya fruit. In this study, a combined metabolomic and transcriptomic analysis was conducted to reveal the possible mechanism of the ripening disorder caused by unsuitable 1-MCP in papaya. A total of 203 differential accumulated metabolites (DAMs) were identified in the metabolome analysis. Only 24 DAMs were identified in the control (CK) vs. the 1-MCP 2 h group, and they were primarily flavonoids. Ninety and 89 DAMs were identified in the CK vs. 1-MCP 16 h and 1-MCP 2 h vs. 1-MCP 16 h groups, respectively, indicating that long-term 1-MCP treatment severely altered the metabolites during fruit ripening. 1-MCP 16 h treatment severely reduced the number of metabolites, which primarily consisted of flavonoids, lipids, phenolic acids, alkaloids, and organic acids. An integrated analysis of RNA-Seq and metabolomics showed that various energy metabolites for the tricarboxylic acid cycle were reduced by long-term treatment with 1-MCP, and the glycolic acid cycle was the most significantly affected, as well as the phenylpropane pathway. These results provide valuable information for fruit quality control and new insight into the ripening disorder caused by unsuitable treatment with 1-MCP in papaya.

## 1. Introduction

Papaya (*Carica papaya* L.), one of the important economic crops in the tropics and subtropics, has high nutritional, economic, and medicinal value [1], with a silky texture and richness in sugar, protein, amino acids, soluble calcium, and multiple vitamins [2,3]. Papaya is a typical climacteric fruit that ripens rapidly after harvest and easily softens and rots during storage and transportation, resulting in a short shelf life and large losses [4,5].

1-methylcyclopropylene (1-MCP) has been widely used in the postharvest preservation of fruits and vegetables and proven to effectively delay fruit senescence and maintain fruit quality [6,7,8]. As an effective inhibitor of the ethylene receptor, 1-MCP irreversibly binds to the receptor, thereby inhibiting the transduction of ethylene signal, which can effectively delay fruit ripening and softening [9,10]. Studies have shown that treatment with 1-MCP can effectively prolong the storage and shelf life of various climacteric and non-climacteric fruits [11,12]. However, there are many problems with the practical applications of 1-MCP. For example, the treatment of 1-MCP on apple fruit reduces the production of many volatile alcohols and esters and results in abnormal fruit softening and coloring [13], and unsuitable treatment with 1-MCP causes uneven coloring in bananas [14,15]. In papaya fruit, studies have shown that treatment with the appropriate concentration of 1-MCP when fruit with more than 25% skin yellow significantly delayed the ripening of fruit and extended its shelf life, without an unfavorable impact on fruit flavor and taste [9,16]. However, unsuitable uses of 1-MCP, such as a high concentration of 1-MCP, a long-term treatment time, or fruit with low maturation [16,17], will cause a ripening disorder of papaya, in which the fruit fails to ripen and soften completely, known as “rubbery texture” [18,19].

To date, several studies have shown that the ripening disorder caused by treatment with 1-MCP may be owing to the effects of 1-MCP on ethylene-related genes, including the regulation, signal transduction and receptor genes of ethylene synthesis [20,21], and its further impact on fruit ripening and softening-related enzymes and genes, including pectin methylesterase (PME), polygalacturonase (PG), pectate lyase (PL), β-galactosidase (β-GAL), and other enzymes [22,23]. However, the underlying mechanism still merits further investigation.

During the past few decades, the integration analysis of metabolic with transcriptome data has proven to be an effective way to identify key genes and important metabolic pathways in plants [24]. Increasing amounts of research focus on the molecular mechanisms of transcriptome and metabolomics during the processes of various biological stress, abiotic stress, growth, and development [25,26,27]. Multi-omics is also gradually being applied to the regulation of fruit ripening. Nogueira et al. analyzed the differential proteomics of papaya fruit during the fruit ripening process and obtained 37 significantly different proteins related to sugar metabolism, secondary metabolism, and the synthesis and regulation of ethylene, energy metabolism, and stress responses [28]. A transcriptomics analysis showed that the key genes of lignin and cellulose are differentially expressed during papaya fruit ripening, as are key genes in the process of ethylene signal transduction involved in the fruit softening disorder [16]. Increasing numbers of studies have also shown that metabolites are closely related to the ripening, senescence, and disease response of postharvest fruit [29,30]. It has been reported that the contents of amino acids, flavonoids, lipids, and terpenoids change significantly during the ripening process of cherry fruit, and, in particular, the content of flavonoids was closely related to the decline in antioxidant capacity of ripening cherries [31]. It was found that three UDP-glucose flavonoids were involved in the accumulation of anthocyanins and the color changes during the ripening process of winter jujube [32]. Giné-Bordonaba et al. showed that treatment with 1-MCP reduced the occurrence of superficial scald disease during pear storage. The levels of transcription and metabolism have undergone tremendous changes after treatment, among which the biosynthesis of long-chain fatty acids plays a critical role in the disease response [33].

The ripening disorder of papaya fruit is also closely related to metabolites, but the related mechanism remains unclear. Previous work showed that phenylpropane metabolism is important for the regulation of growth and development in plants [34]. Lignin, flavonoids, and phenolics are closely related to the physiological processes of cell differentiation, cell wall structure, and coloring pigments during fruit ripening and softening [35,36]. Flavonoids are involved in the change in color and antioxidation activity of fruit during the ripening and softening process [37]. Our previous work showed that lignin and cellulose are closely related to the papaya ripening disorder by affecting the degradation of the cell wall [16]. In this study, the combined analysis of transcriptome and metabolome were utilized to reveal the possible mechanism of ripening disorder caused by unsuitable treatment with 1-MCP, which would provide a theoretical guide for the application of 1-MCP in the preservation of papaya, as well as other fruit with ripening disorders.

## 2. Results

### 2.1. Overview of the Fruit Metabolome Profiling under Different Conditions

Our previous work showed that short-term treatment with 1-MCP could delay papaya fruit ripening and softening, but long-term treatment with 1-MCP caused the fruit softening disorder known as rubbery texture [16]. As shown in the previous work (https://link.springer.com/article/10.1186/s12870-019-1904-x/figures/1) [16], all fruit under different conditions could turn yellow completely during storage. Fruit firmness decreased rapidly under control and short-term treatment of 1-MCP and to a low level at the end of storage. However, fruit under long-term treatment with 1-MCP kept a high firmness during the whole storage [16]. The metabolome analysis was conducted to investigate the possible mechanism of softening disorder caused by unsuitable 1-MCP treatment in more detail. To compare the differences of metabolite compositions of papaya fruit under different treatments (1-MCP treated with 2h, 1-MCP treated with 16h and without 1-MCP), the samples of papaya flesh on the 6th day after treatment were subjected to an LC-ESI-MS/MS analysis. The repeatability of metabolite detection can be evaluated using an overlapping analysis of the total ion current (TIC) using the quality control samples. As shown in Figure S1, the retention times and peak intensities of metabolites were consistent in the quality control samples and experimental samples, indicating that the signal and instrument were stable in our experiment, which provided the repeatability and reliability of metabonomic data for the analysis. In addition, the sample repetition correlation graph showed that the sample reproducibility is good, and the metabolome data can be used for subsequent analyses (Figure S2).

**Figure 1:**
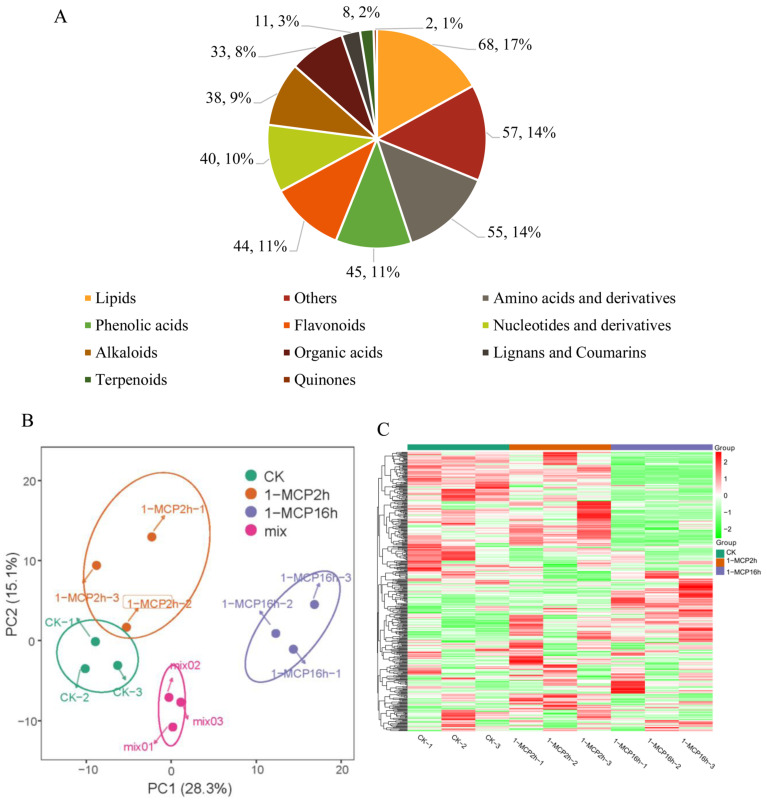
Overall qualitative and quantitative analysis of the metabolomics data. (**A**) Component analysis of the identified metabolites. (**B**) Principal component analysis (PCA) of the three group samples and quality control samples; the *x*-axis represents the first principal component, and the *y*-axis represents the second principal component. (**C**) Clustering heat map of the metabolites. The metabolite content data were normalized. CK, 6 days after treatment (DAT) under the control conditions. 1-MCP 2 h, 6 DAT under the 1-MCP 2 h treatment; 1-MCP 16 h, 6 DAT under the 1-MCP 16 h treatment.

In this study, a total of 401 distinct annotated metabolites were detected in the samples, which were grouped into 11 classes (Figure 1A, Table S1). The majority were lipids (68, 16.9%), amino acids and derivatives (55, 13.17%), phenolic acids (45, 11.2%), followed by flavonoids (44, 10.9%), nucleotides and derivatives (40, 9.9%), alkaloids (38, 9.4%) and other metabolites (Figure 1A).

Metabolite profiles of papaya fruit were then subjected to a PCA analysis. The score plots of PCA (Figure 1B) showed an obvious separation between different 1-MCP treatments and the control. The first two principal components (PC1 and PC2) of the metabolites of these nine samples accounted for 27.98% and 14.99% of the total metabolites, respectively. As shown in Figure 1B, a significant separation between 1-MCP 16 h and control in PC1 was apparent, but there was no obvious separation of PC2. No significant separation of PC1 was observed between 1-MCP 2 h and the control, but an obvious separation of PC2 was observed (Figure 1B). These results indicate that there was a large degree of variance in the metabolites between 1-MCP 16 h and the control group. A hierarchical heatmap clustering analysis of the samples showed that all the biological replicates were grouped together, indicating good quality and high reliability of the metabolome data (Figure 1C). Interestingly, the samples of papaya with 1-MCP 16 h differed significantly from those of the control and 1-MCP 2 h, while the difference between control and 1-MCP 2 h was relatively small. The clustering heat maps showed that the short-term 1-MCP treatment repressed the production of some metabolites, while the long-term 1-MCP-treated samples dramatically inhibited the production of many metabolites and induced the production of others. In summary, the cluster heat map showed that the metabolite profiles of samples treated with 1-MCP for different durations differed dramatically.

### 2.2. Identification of Differentially Accumulated Metabolites (DAMs)

**Figure 2:**
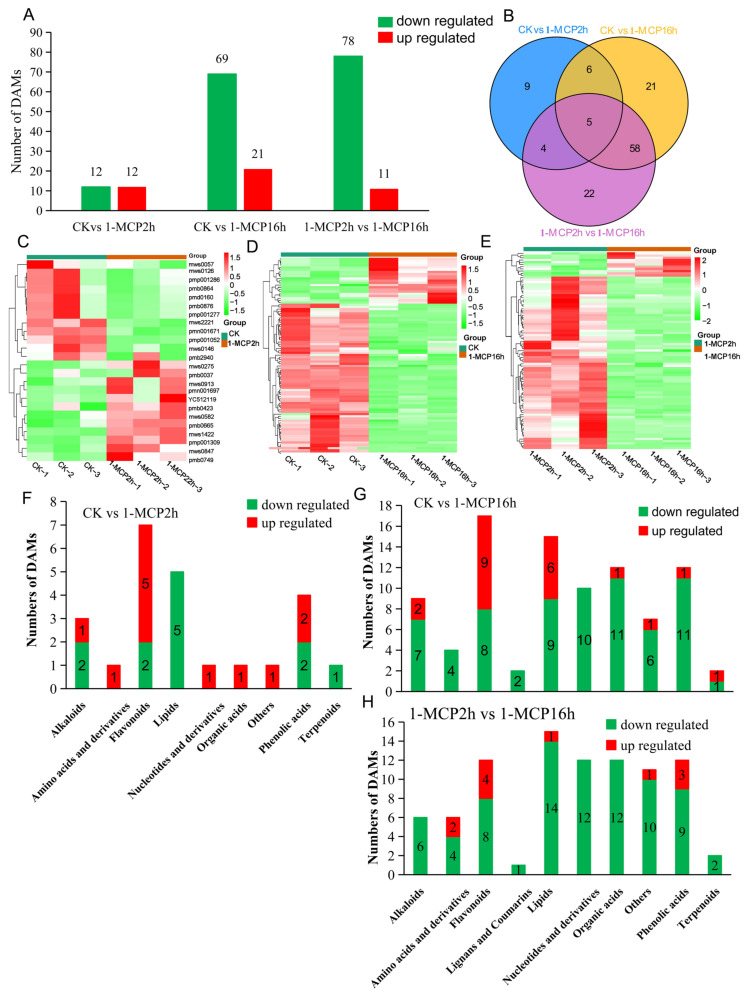
Differentially accumulated metabolites (DAMs) analysis under different 1-MCP treatments. (**A**) The numbers of metabolites with differences in relative abundance that were up-regulated (red) and down-regulated (green) in the papaya fruit of CK vs. 1-MCP 2 h group, CK vs. 1-MCP 16 h group, and 1-MCP 2 h vs. 1-MCP 16 h group. (**B**) Venn diagram showing the shared and unique DAMs among different comparison groups. (**C**–**E**) Heat map of the DAMs in different comparison groups: CK vs. 1-MCP 2 h (**C**), CK vs. 1-MCP 16 h (**D**), and 1-MCP 2 h vs. 1-MCP 16 h (**E**). (**F**–**H**) Number of DAMs in different categories of the different comparison groups: CK vs. 1-MCP 2 h (**F**), CK vs. 1-MCP 16 h (**G**), and 1-MCP 2 h vs. 1-MCP 16 h (**H**).

We used the Orthogonal Partial Least Squares Discriminant Analysis (OPLS-DA) and R software to identify the differential metabolites and verify the OPLS-DA 200 times, which showed that the model was meaningful and can be screened for differential metabolites based on their VIP. The DAMs were defined as those metabolites with a fold change ≥2 or a fold change ≤0.5 and a VIP ≥1 between the control (CK) versus 1-MCP 2 h, CK versus 1-MCP 16 h, or 1-MCP 2 h versus 1-MCP 16 h (*p* < 0.05). In total, 203 DAMs were identified, of which 24, 90, and 89 were identified in the group of CK versus 1-MCP 2 h, CK versus 1-MCP 16 h, and 1-MCP 2 h versus 1-MCP 16 h, respectively (Figure 2A). For the CK vs. MCP 2 h group, 12 of 24 (50%) were up-regulated, and another 50% were down-regulated. Of the 90 metabolites differentially accumulated in the CK versus 1-MCP 16 h, 21 (23.3%) and 69 (76.7%) metabolites were up-regulated and down-regulated, respectively. Of the 89 DAMs identified between 1-MCP 2 h versus 1-MCP 16 h, 11 (12.4%) and 78 (87.6%) metabolites were up-regulated and down-regulated, respectively (Figure 2A; Table S2). Five of these 203 DAMs were commonly shared by the CK vs. 1-MCP 2 h, CK vs. 1-MCP 16 h, and 1-MCP 2 h vs. 1-MCP 16 h, which were phytosphingosine, nicotinic acid methyl ester (methyl nicotinate), (-)-epiafzelechin,1-o-β-d-glucopyranosyl sinapate and penstemonoside, alkaloids, flavonoids, phenolic acids, and terpenoids. In addition, 9, 21, and 22 DAMs specifically accumulated in the CK vs. 1-MCP 2 h, CK vs. 1-MCP 16 h, and 1-MCP 2 h vs. 1-MCP 16 h, respectively (Figure 2B, Table S3). Among these DAMs, only 11 were shared by the group of CK vs. 1-MCP 2 h and CK vs. 1-MCP 16 h. Most of the DAMs specifically accumulated in the 1-MCP 16 h samples, validated by the group of CK vs. 1-MCP 16 h and 1-MCP 2 h vs. 1-MCP 16 h, which were shared by 63 DAMs (Figure 2B).

The DAMs were also shown by volcano plots in Figure S2, which presented the significant differences between the CK vs. 1-MCP 2 h, CK vs. 1-MCP 16 h, or 1-MCP 2 h vs. 1-MCP 16 h (Figure S3A–C). Of these DAMs, flavonoids (29%), lipids (21%), phenolic acids (17%), and alkaloids (13%) accounted for a large proportion in the CK vs. 1-MCP 2 h group (Figure S3D). Of the DAMs in CK vs. 1-MCP 16 h group, flavonoids (19%), lipids (17%), phenolic acids (13%), organic acids (13%), and nucleotides and derivatives (11%) were the most prevalent (Figure S3E). Lipids (17%), phenolic acids (14%), nucleotides and derivatives (14%), flavonoids (13%), organic acids (13%), and other compounds (12%) were shown to be differentially accumulated in the 1-MCP 2 h vs. 1-MCP 16 h group (Figure S3F).

The hierarchical cluster analysis obviously showed different patterns of accumulation of DAMs in different groups. One cluster of metabolites (12) showed a high level in the control samples but a low level in the samples treated with 1-MCP 2 h. However, another cluster of metabolites accumulated less in the control samples but was significantly enhanced by treatment with 1-MCP 2 h (Figure 2C). In the group of control vs. 1-MCP 16 h, a small cluster of metabolites were present at a low level in the control samples but accumulated to a significantly higher level in the samples treated with 1-MCP 16 h. A large cluster of metabolites was found at high levels in the control samples but was dramatically reduced by the 1-MCP 16 h treatment (Figure 2D). More metabolites were repressed by the 1-MCP 16 h treatment in the comparison of 1-MCP 2 h vs. 1-MCP 16 h (Figure 2D), which may be related to the ripening disorder caused by the 1-MCP 16 h treatment.

The DAMs identified in this study primarily included alkaloids, flavonoids, lipids, organic acids, phenolic acids, terpenoids, amino acids and derivatives, lignans and coumarins, and nucleotides and derivatives. As shown in Figure 2F, 1-MCP 2 h reduced the accumulation of lipids, terpenoids and portions of the alkaloids, flavonoids, and phenolic acids but induced the accumulation of amino acids and derivatives, nucleotides and derivatives, organic acids, and others, as well as the portion of alkaloids, flavonoids, and phenolic acids (Figure 2F). Long-term treatment with 1-MCP (1-MCP 16 h) dramatically reduced all these classes of metabolites (Figure 2G). In the comparison group of 1-MCP 2 h vs. 1-MCP 16 h, more metabolites were reduced, particularly for the alkaloids, lipids, organic acids, phenolic acids, amino acids and derivatives, lignans and coumarins, and nucleotides and derivatives (Figure 2H).

### 2.3. Functional Annotation and KEGG Enrichment Analysis of DAMs

The 203 DAMs were mapped to the KEGG database (Table S4). For the CK vs. 1-MCP 2 h group, the results showed that the metabolites only mapped to “metabolism,” and most of them mapped to “metabolic pathways” and “biosynthesis of secondary metabolites” pathways (Figure S4A). For the CK vs. 1-MCP 16 h comparison group, the “metabolism” classification was also the one that was mapped the most frequently. The “metabolic pathways,” “biosynthesis of secondary metabolites,” “purine metabolism,” and “flavonoid biosynthesis” were mapped the most frequently. Some of the metabolites were also classified into “genetic information processing” or “environmental information processing,” which are “aminoacyl-tRNA biosynthesis” and “ABC transporters,” respectively (Figure S4B, Table S5). Similar results were observed for the 1-MCP 2 h vs. 1-MCP 16 h comparison group, and most of the metabolites were also classified into “metabolic pathways,” “biosynthesis of secondary metabolites,” “purine metabolism,” and “flavonoid biosynthesis,” as well as additional “glycerolipid metabolism” (Figure S4C). These results indicated that metabolites in the “metabolic pathways” and “biosynthesis of secondary metabolites” pathway were related to fruit ripening, and metabolites in the “metabolic pathways,” “biosynthesis of secondary metabolites,” “purine metabolism,” “flavonoid biosynthesis,” “aminoacyl-tRNA biosynthesis,” and “ABC transporters” may be related to the fruit ripening disorder.

**Figure 3:**
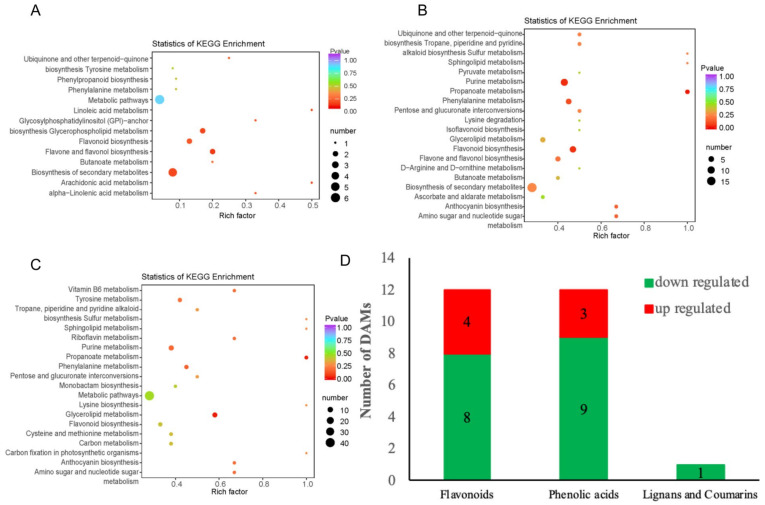
KEGG pathway enrichment analysis of DAMs. (**A**) Pathway enrichment in the CK vs. 1-MCP 2 h group; (**B**) pathway enrichment in the CK vs. 1-MCP 16 h group; (**C**) pathway enrichment in the 1-MCP 2 h vs. 1-MCP 16 h group. The *x*-axis represents the enrichment factor, while the *y*-axis represents the enrichment pathway. The dot sizes represent the number of differentially enriched metabolites. The statistical analysis of the pathway enrichment was performed using Fisher’s exact test. (**D**) Phenylpropanoid metabolites of DAMs in the 1-MCP 2 h vs. 1-MCP 16 h group. DAMs, differentially accumulated metabolites; CK, 6 DAT under the control condition; 1-MCP 2 h, 6 DAT under the 1-MCP 2 h treatment; 1-MCP 16 h, 6 DAT under the 1-MCP 16 h treatment.

The KEGG enrichment analysis was conducted to further investigate the ripening disorder-related metabolites (Table S6). For the CK vs. 1-MCP 2 h group, the terms of ‘biosynthesis of secondary metabolites,’ ‘flavone and flavonol biosynthesis,’ ‘flavonoid biosynthesis,’ and ‘glycerophospholipid metabolism’ were significantly enriched (Figure 3A). The terms ‘propanoate metabolism,’ ‘purine metabolism,’ ‘flavonoid biosynthesis,’ ‘phenylalanine metabolism,’ ‘anthocyanin biosynthesis,’ and ‘amino sugar and nucleotide sugar metabolism’ were enriched in the CK vs. 1-MCP 16 h group (ripening disorder group) (Figure 3B). For the 1-MCP 2 h vs. 1-MCP 16 h comparison group, the DAMs were most significantly enriched in the terms ‘propanoate metabolism,’ ‘glycerolipid metabolism,’ ‘phenylalanine metabolism,’ and ‘purine metabolism’ (Figure 3C), indicating that these pathways may be closely related to the papaya fruit ripening disorder.

In the CK vs. 1-MCP 2 h group, the terms ’flavone and flavonol biosynthesis’ were mostly enriched, indicating that ’flavone and flavonol biosynthesis’ plays a key role in the 1-MCP 2 h treatment during the ripening of papaya fruits. Interestingly, we found that the most up-regulated metabolites were classified to flavonoids among the DAMs of CK vs. 1-MCP 2 h, which are shown in Table S2, and the down-regulated DAMs were primarily lipids. These data indicated that the short-term 1-MCP treatment delays the ripening and senescence of papaya fruits by inhibiting the metabolism of lipid-based metabolites and inducing the production of flavonoid-based metabolites.

For the CK vs. 1-MCP 16 h group, ‘propanoate metabolism’ was the predominantly significantly enriched term, which may be closely related to the formation of ripening disorder of papaya fruits caused by the long-term treatment with 1-MCP. Among the 21 up-regulated DAMs, 9 were flavonoids (42.9%), and 6 were lipids (28.6%) (Figure 2G). There were 69 kinds of down-regulated DAMs, with phenolic acids (15.9%) and organic acids (15.9%) predominating. Moreover, nucleotides and derivatives, lipids, flavonoids, and alkaloids were also down-regulated (Figure 2G). The changes in the content of these metabolites may play an important role in cell structure regulation, edible value, and signaling. These results showed that the 1-MCP 16 h treatment led to severe inhibition of the metabolic process of multiple metabolites in the ripening process of papaya fruit and induced the increase in some metabolites of flavonoids and lipids, which further prevented the ripening of papaya fruit. 

The lignin synthesis pathway and the flavonoid metabolism pathway are important branches of the phenylpropane pathway. We found that various DAMs are involved in the phenylpropane metabolism pathway, including flavonoids, phenolic acids and lignans and coumarins (Table S2). As shown in Figure 3D, most of the flavonoids, phenolic acids and lignans and coumarins were down regulated by long-term treatment with 1-MCP compared with the short-term treatment with 1-MCP, indicating that these metabolites are important for the fruit softening process and are critical components for the development of ripening disorder.

### 2.4. Different Accumulation Pattern of DAMs under Differing Conditions

**Figure 4:**
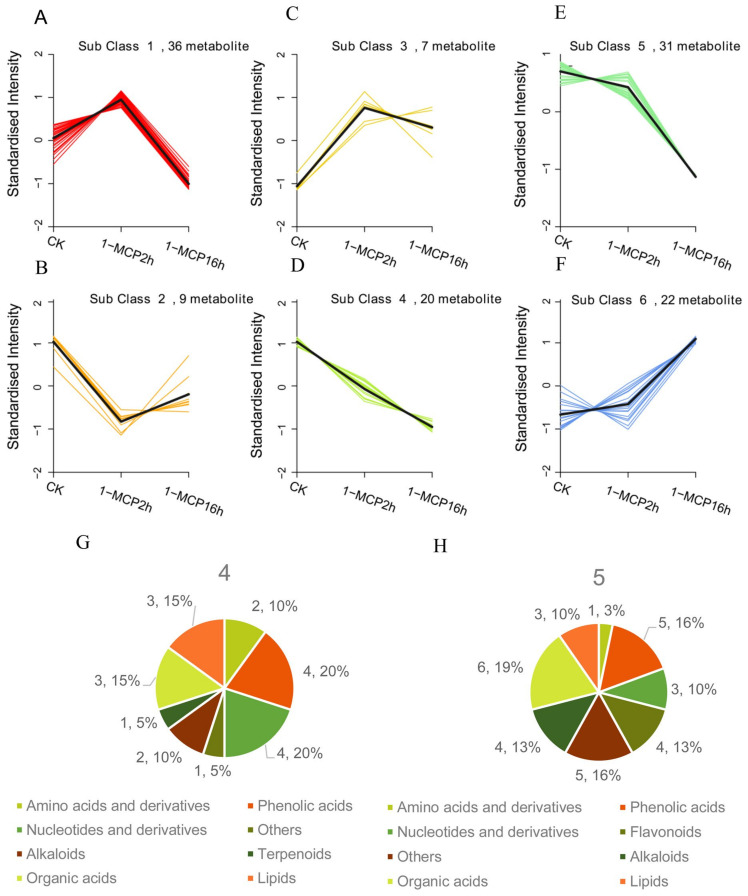
Different patterns of accumulation affected by different 1-MCP treatments. (**A**–**F**) K-means clustering of the DAMs into six clusters. The x-axis depicts nine samples from three different treatments, and the y-axis depicts the standardized intensity per metabolite. The black line showed the change trends of DAMs under different conditions in the k-means clustering. (**G**) Component analysis of DAMs in class 4. (**H**) Component analysis of DAMs in class 5.

As shown in Figure 4, the pattern of accumulation of the DAMs could be grouped into six clusters (Figure 4A–F, Table S7). Among these classes, the DAMs in class 4 (Figure 4D) and class 5 (Figure 4E) decreased with the increase in the duration of the 1-MCP treatments, which appeared to be more closely related to the fruit ripening and softening disorder. The DAMs in these two classes were primarily phenolic acids, flavonoids, organic acids, nucleotides and derivatives, and alkaloids (Figure 4G,H, Table S7), indicating that these metabolites were important for fruit ripening and softening.

### 2.5. Integrated Analysis of RNA-Seq and Metabolomics Data

**Figure 5:**
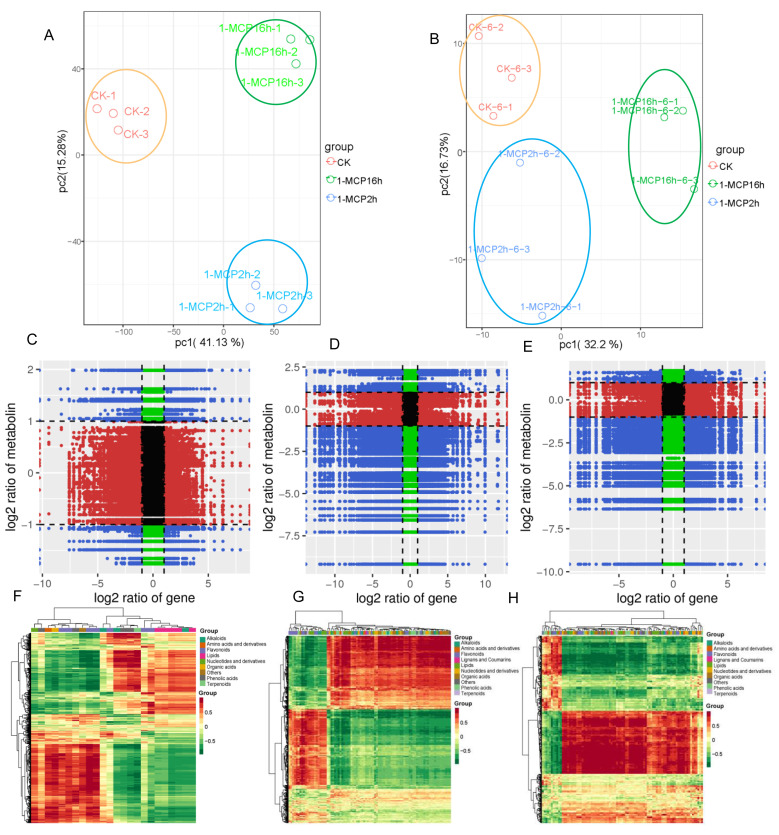
Integration analysis of DAMs and DEGs. (**A**,**B**) PCA analysis of transcriptome (**A**) and metabolome (**B**) data; (**C**–**E**) quadrant diagrams representing the association of metabolomic and transcriptomic variation; (**F**–**H**) correlation coefficient clustering heat map of different comparison group: CK vs. 1-MCP 2 h group (**F**); CK vs. 1-MCP 16 h group (**G**); 1-MCP 2 h vs. 1-MCP 16 h group (**H**).

The overview of RNA-Seq quality was comparable to that described by Zhu et al. (2019) [16]. Further analysis for the comparison of CK vs. 1-MCP 2 h, CK vs. 1-MCP 16 h and 1-MCP 2 h vs. 1-MCP 16 h were conducted in this study. As shown in Figure 5A,B, different treatments group together and were separated from other groups when a PCA analysis of the RNA-Seq and metabolomic data was conducted. For the CK vs. 1-MCP 2 h treatment, 1154 up-regulated and 1430 down-regulated differentially expressed genes (DEGs) were identified. The DEGs increased under long-term treatment with 1-MCP (CK vs. 1-MCP 16 h), in which 1891 up-regulated and 2121 down-regulated DEGs were identified in the CK vs. 1-MCP 16 h group. In the 1-MCP 2 h vs. 1-MCP 16 h group, there were 522 up-regulated genes and 535 down-regulated genes, which may be closely related to the papaya fruit ripening disorder. The GO and KEGG enrichment analyses can also be found in Zhu et al. (2019) [16].

**Figure 6:**
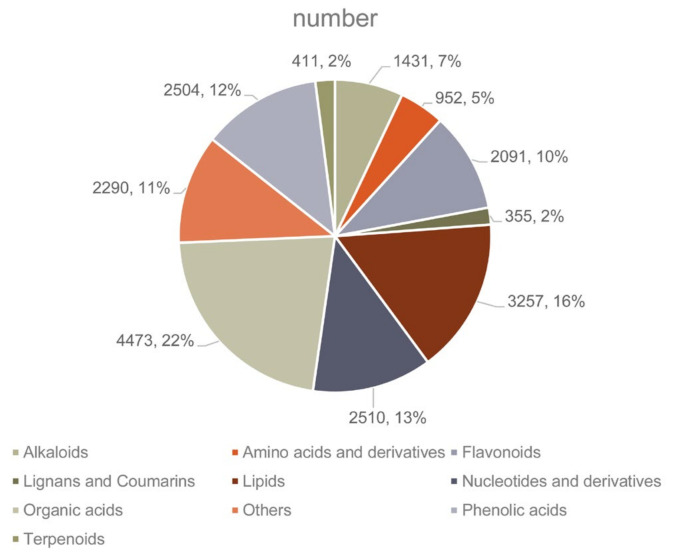
The number of down-regulated DEGs corresponding to the DAMs in the correlation analysis of 1-MCP 2 h vs. 1-MCP 16 h group.

An integrated analysis between the DEGs and DAMs was conducted. A KEGG analysis showed that 12 pathways were enriched in both DAMs and DEGs in the CK vs. the 1-MCP 2 h group (Figure S5A). However, 39 pathways were enriched by DAMs and DEGs in the CK vs. 1-MCP 16 h group, and more significant *p*-values were observed (Figure S5B), particularly for the phenylalanine metabolism, propanoate metabolism, phenylpropanoid biosynthesis, ubiquinone, and other terpenoid-quinone biosynthesis pathway. More pathways (46) were significantly enriched in the 1-MCP 2 h vs. 1-MCP 16 h comparison, particularly for glycerolipid metabolism, propanoate metabolism, phenylalanine metabolism, purine metabolism, and riboflavin metabolism (Figure S5C). Correlation analysis of the DEGs and DAMs detected in each group was conducted using the cor program in R to calculate the PCC. The DEGs and DAMs with a correlation greater than 0.8 were selected and shown in the nine quadrant diagrams in Figure 5. The black dotted lines divided each graph into nine quadrants, which correlated differently between the DEGs and DAMs. For quadrant 1 and 2, it showed the up-regulated metabolites and down-regulated genes and up-regulated metabolites and unchanged genes, respectively. The up-regulated metabolites and up-regulated genes were displayed in quadrant 3, and the unchanged metabolites and down-regulated genes are shown in quadrant 4. Quadrant 5 showed the unchanged metabolites and unchanged genes. Quadrant 6 showed the unchanged metabolites and up-regulated genes. The down-regulated metabolites and down-regulated genes are presented in quadrant 7; the down-regulated metabolites and unchanged genes are shown in quadrant 8, and the down-regulated metabolites and up-regulated genes are presented in quadrant 9. As shown in Figure 5C, there was a large portion of metabolites and genes in the CK vs. 1-MCP 2 h group that were not correlated, but there was a significantly lower portion of them in the CK vs. 1-MCP16 and 1-MCP 2 h vs. 1-MCP 16 h groups (Figure 5C–E). The important DAMs and DEGs are presented in quadrants 3 and 7 of 1-MCP 2 h vs. 1-MCP 16 h group, which positively correlated and showed similar consistently changing patterns (Table S8). In contrast, the DAMs and DEGs displayed in quadrant 1 and quadrant 9 were negatively correlated and showed opposite change patterns (Table S9). These results also confirmed and are presented in Figure 5F–H, in which a large portion of the red section displayed a positive correlation between the DEGs and DAMs in CK vs. 1-MCP16 and 1-MCP 2 h vs. 1-MCP 16 h groups, indicating that they may be closely related to fruit quality and the ripening disorder. Notably, in quadrant 7 of 1-MCP 2 h vs. 1-MCP 16 h group, those genes and metabolites were both significantly repressed by treatment with 1-MCP and are closely related to ripening in papaya fruit. Among them, 4473, 3257, 2504, 2091, and 2510 DEGs were related to the DAMs of lipids, phenolic acids, flavonoids and nucleotides, and derivatives metabolites, respectively (Figure 6, Table S8).

### 2.6. DEGs and DAMs Analyses under Long-Term 1-MCP Treatment

**Figure 7:**
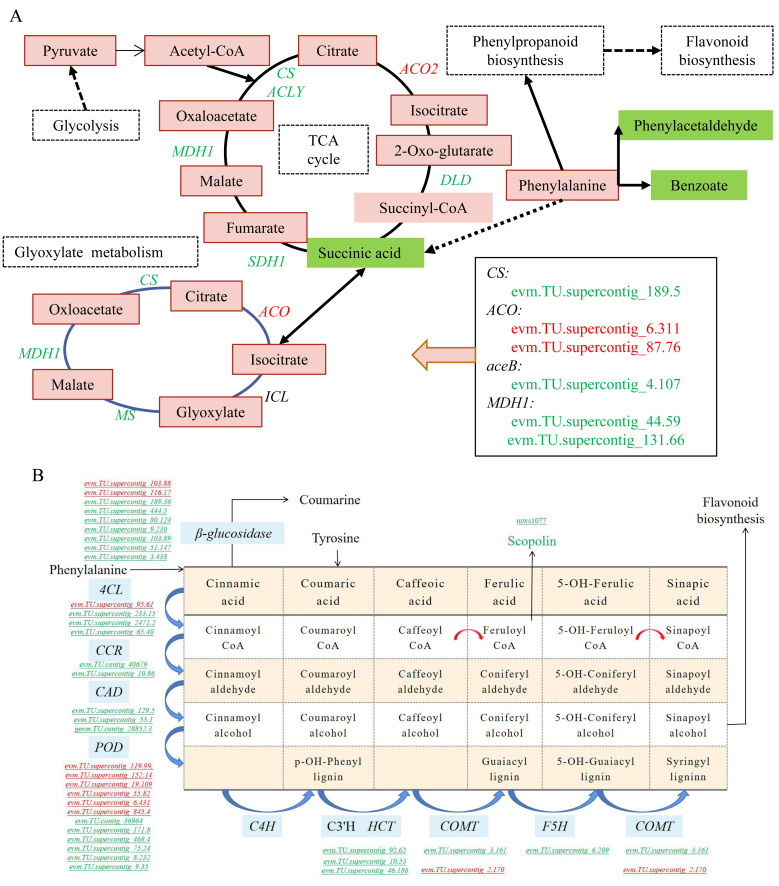
Important pathways affected by long-term 1-MCP treatment. (**A**) Energic supply pathways for certain important DAMs and DEGs under long-term 1-MCP treatment; (**B**) biosynthetic pathway of phenylpropanoids. Red and green represent increased and decreased metabolites and transcripts, respectively.

Based on the analysis of DAMs and DEGs and the correlation analysis, we found that long-term 1-MCP significantly inhibited the basal metabolism and secondary metabolites of papaya, including the glyoxylic acid cycle, tricarboxylic acid (TCA) cycle, pyruvate metabolism, and other processes (Figure 7A), which could possibly account for the ripening disorder of papaya during storage. Citrate synthase (CS), isocitrate dehydrogenase (IDH), and α-ketoglutarate dehydrogenase complexes are the rate-limiting enzymes in the TCA cycle. A large amount of nicotinamide adenine dinucleotide (NADH) is produced in the reaction processes of CS, aconitase (ACO), IDH, oxoglutarate dehydrogenase complex (ODC), succinate dehydrogenase (SDH), and malate dehydrogenase (MDH), involved in the TCA cycle [38]. We found that the expression of *CS, DLD (dihydrolipoamide dehydrogenase), ACLY (ATP citrate (pro-S)-lyase), SDH1 (succinate dehydrogenase (ubiquinone) flavoprotein subunit),* and *MDH* were severely inhibited by 1-MCP 16 h. The various organic acids that may be involved in the energy metabolism pathways and centered on the TCA cycle (succinic acid) were reduced by 1-MCP 16 h, as well as in the glyoxylic acid cycle pathway. In addition, the downstream metabolic pathways of the TCA cycle, including fatty acid synthesis and amino acid synthesis, were inhibited by 1-MCP 16 h (Figure 7A).

We also found that “phenylpropanoid biosynthesis” was markedly affected, and the detailed network of this pathway was mapped (Figure 7B). In this pathway, we identified 36 DEGs and 1 DAM. The 36 structural genes included 4 *4-coumarate--CoA ligase (4CL)*, 2 *cinnamoyl-CoA reductase (CCR)*, 3 *cinnamyl-alcohol dehydrogenase (CAD)*, 1 *5-O-(4-coumaroyl)-D-quinate 3’-monooxygenase (C3’H)*, 2 *shikimate O-hydroxycinnamoyltransferase (HCT)*, 1 *caffeic acid 3-O-methyltransferase (COMT)*, 1 *ferulate-5-hydroxylase (F5H)*, 9 *β-glucosidase*, and 12 *peroxidase (POD)*, which are the major structural genes in the phenylpropanoid biosynthesis pathway. In addition, *E2.1.1.104* (*caffeoyl-CoA O-methyltransferase*) was identified. In particular, most of these key genes in this pathway were down-regulated.

## 3. Discussion

Papaya fruit is regarded as a good source of nutrients, including provitamin A, carotenoids, vitamin B, vitamin C, lycopene, dietary minerals, and fiber, which greatly benefitss human health, as well as having various medicinal uses [39]. However, as a typical climacteric fruit, papaya fruit is subject to rapid ripening and a deterioration in quality after harvest [23,40]. In our previous work, we showed that treatment with 1-MCP and low temperatures could effectively delay fruit ripening and maintain fruit quality [16,23]. However, low temperature and unsuitable treatment with 1-MCP will cause fruit ripening disorder, which results in serious economic and social consequences to the papaya industry [16,41]. In this study, the combined analyses of transcriptomes and metabolomes were utilized to reveal the possible mechanism of the ripening disorder caused by unsuitable treatment with 1-MCP. The metabolite profiling is critical to discover biomarkers during the complex processes of fruit ripening and senescence. Various studies employ the metabolome and transcriptome tools to study the key genetic and molecular factors that affect and regulate fruit ripening and senescence, such as those in peach [42], tomato [43,44], pear [33], banana [45], mango [46], and other fruits. There are also limited reports on the study of metabolites in papaya. Gogna et al. analyzed the metabolic profiles in papaya leaves and seeds, aiming to reveal their phytomedicinal constituents using NMR and UPLC-ESI–MS experiments and demonstrated that papaya extracts possessed activities of high antioxidants, such as phenolics and flavonoids [47]. Harini et al. found that the papaya leaf extract is rich in flavonoids, fatty acids, alkaloids, and phenolics and assessed their antimicrobial, anticarcinogenic, antioxidant, and insecticidal properties [48]. Wu et al. showed that the metabolites related to aroma are the major metabolic changes that take place in the papaya fruit peel and substantially contribute in response to cold stress [49]. Metabolomics and proteomics analyses showed that the altered metabolism of fatty acids and amino acids during fruit ripening could result in the synthesis of different types of volatile compounds and affect the sensorial quality of papaya fruit [50]. In this study, our results showed that treatment with 1-MCP altered the metabolites during fruit ripening, particularly for the long-term treatment with 1-MCP. Treatment with long-term 1-MCP severely reduced the metabolites, which primarily included flavonoids, lipids, phenolic acids, alkaloids, and organic acids, indicating that unsuitable treatment with 1-MCP severely impaired the fruit antioxidant system and pharmacological activity. The integrated analysis of RNA-Seq and the metabolome showed that the TCA cycle, the synthesis of various amino acids, sugars, and lipids, as well as secondary metabolic pathways, were inhibited by treatment with 1-MCP, such as the phenylpropane pathway, which may be closely related to the fruit ripening disorder.

The metabolism of lipids, particularly those in the membrane, is one of several important biochemical manifestations of cellular senescence in plants [51]. Fruit softening is primarily owing to the deterioration of cell wall and membrane. The deterioration of the membrane is an early event of senescence that engenders increased permeability, a decreased function of key membrane proteins, and the loss of ionic gradients [52,53]. The damage in functional and structural integrity to membranes may be owing to phospholipid metabolism. It has been demonstrated that the loss of lipid phosphate has been detected in senescent flower petals, leaves, cotyledons, and ripening fruit [54,55]. In this study, as shown in Figure 2, both the treatment with 1-MCP reduced the content of lipids by repressing their metabolism, and the long-term treatment with 1-MCP resulted in more severe inhibition, indicating that the metabolism of lipids is important for the fruit ripening process. In 1-MCP 2 h vs. 1-MCP 16 h, most of the DAMs are lipids and are repressed by long-term treatment with 1-MCP, indicating that the metabolism of lipids should play a role in the fruit softening disorder (Figure 2H). These results echo our previous studies that have shown that treatment with 1-MCP maintains the integrity of fruit cell walls and delays the fruit ripening process. Additionally, long-term treatment with 1-MCP inhibits the degradation of cell walls and causes fruit ripening disorder [23]. It also proved that the metabolism of lipids, particularly that of the membrane lipids, was inhibited by long-term treatment with 1-MCP, which can partially account for the softening disorder.

Organic acids can support numerous and diverse functions in plants. For example, organic acids are an important indicator of fruit flavor, as well as the respiratory substrate during fruit development and ripening. Various organic acids are the intermediates of the TCA cycle [56]. As shown in Figure 2, only L-(-)-malic acid differentially accumulated between the control and 1-MCP 2 h and was up-regulated by treatment with 1-MCP (Figure 2F). However, the contents of organic acids were severely reduced by long-term treatment with 1-MCP, and 11 kinds of organic acids were down-regulated by 1-MCP 16 h. All of the 12 DAMs of organic acids were down-regulated between 1-MCP 2 h vs. 1-MCP 16 h, indicating that these organic acids may be critical for fruit softening. Since the samples were taken on the 6th day, which was 6 days ahead of the formation of rubbery texture phenotype, we hypothesized that long-term 1-MCP severely reduced the organic acids, which served as the intermediates of the TCA cycle and then reduced the energy supply for fruit ripening. The integrated analysis of metabolome and transcriptome found that multiple energy metabolism pathways, such as the TCA cycle, and downstream secondary metabolic pathways, such as phenylpropane metabolism, were inhibited, which may account for ripening disorder caused by long-term treatment with 1-MCP.

Energy is the basis of life activities. In recent years, some studies have shown that the physiological disorders of fruits, including chilling damage and browning, may also be closely related to the reduction in levels of cellular energy [57,58,59]. The energy status is closely related to the ripening and senescence of fruits and vegetables after harvest [60,61]. 1-MCP inhibits ethylene-dependent reactions and regulates the energy supply to maintain the quality of various fruits, including nectarine [62] and “Jonagold” apple [63]. Ali et al. found that in kiwifruit, treatment with 1-MCP can maintain the quality of fruit flavor by improving the TCA cycle and mitochondrial energy metabolism and limiting the accumulation of ethanol [64]. This study also showed that the energy metabolic pathways, such as the TCA cycle and the glyoxylic acid cycle, played a pivotal role in the regulation of papaya ripening disorder caused by long-term treatment with 1-MCP (Figure 7A). The expression of the key rate-limiting enzyme that encodes the CS gene in the TCA cycle was inhibited, and the genes that encoded DLD, ACLY, SDH1, and MDH that are involved in the production of NADH were also inhibited. The CS enzyme is part of the α-ketoglutarate dehydrogenase complex, which regulates the conversion of α-ketoglutarate to succinate, while the DLD, ACLY, SDH1, and MDH enzymes catalyze the TCA cycle pathway to produce NADH, GTP (guanosine triphosphate), or FADH2 (flavin adenine dinucleotide, reduced), which are important substances of the cellular energy system [38,65].

The metabolism of phenylpropanes has important physiological significance in plants, including biotic and abiotic stress responses, environmental adaption, reproduction, and plant development, which is primarily manifested in the changes in the activity of enzymes, the intermediate products, and their transformed products (lignin, flavonoids, phytoalexins, and phenolics) [34,66]. Flavonoids are ubiquitous plant secondary metabolites with various biological functions, including plant coloring and abiotic or biotic stress response, as well as plant growth and development [67]. Flavonoids are derived from the phenylpropanoid pathway, which transforms phenylalanine into 4-coumaroyl-CoA using several crucial enzymes, such as PAL, 4CL, and C4H. Phenolic compounds are important constituents in vegetable foods for their beneficial effects on human health. Phenolic compounds may also play an important role in the protection against reactive oxygen species (ROS) in the plant owing to their redox properties [68]. Previous work has shown that the antioxidative metabolism is important for the potential storage or shelf-life of fruit and susceptibility to various physiological disorders [69]. In this study, 31 phenylpropane metabolites differentially accumulated in CK-6 vs. 1-MCP 16 h (Figure 2G), including phenolic acids, flavonoids, lignans, and coumarins. Twenty-one of the 31 phenylpropanes were down-regulated by 1-MCP 16 h, indicating that phenylpropane metabolites are closely related to the ripening disorder induced by long-term treatment with 1-MCP. The joint analysis of metabolome and transcriptome also showed that the phenylpropane metabolic pathway was severely inhibited, and the genes encoding key enzymes, such as 4CL, CCR, CAD, C4H, C3’H, COMT, and F5H, were inhibited (Figure 7B). Increasing numbers of studies have shown that the phenylpropane pathway plays a vital role in fruit softening [16], color change [32], and the ability to respond to stress [25]. Our previous work also showed that long-term treatment with 1-MCP severely affected the expression of genes in the phenylpropane pathway and maintained higher levels of cellulose and lignin [16,23]. Flavonoids are one of the three major pigments of plants. They contain six categories of flavonoids, including flavonoids, flavonols, and anthocyanins. These are essential substances in the ripening process of plants [32]. Flavanone 3-hydroxylase (F3H) is one of the key enzymes in the metabolic pathway of flavonoids [70]. In the CK vs. 1-MCP 2 h group, genes in the flavonoid metabolism pathway showed a significant differential expression, and the activity of F3H showed a downward-regulated trend. However, in the CK vs. 1-MCP 16 h group, the activities of HCT and C3’H in flavonoid metabolism are inhibited, which may account for the down-regulated flavonoid production in the fruit treated with long-term 1-MCP.

## 4. Materials and Methods

### 4.1. Plant Material and Sampling

Cv. “Suiyou-2” papaya fruit at the color break stage (with their peel color between 5–15% yellow) were harvested at a local commercial orchard in Panyu District of Guangzhou city, China, as in our previous work [16]. The fruit was sorted and selected for uniformity and the absence of defects immediately after harvest. The fruit was then washed with water and immersed in 0.2% (*w*/*v*) hypochloride solution for 10 min. The fruit was removed and soaked in a mixture of iprodione (Jiangsu Kuaida Agrochemical Co., Ltd., Dafeng District, China) and prochloraz (Jiangsu Huifeng Agrochemical Co., Ltd., Dafeng District, China) at 500 mg mL^−1^ concentration for 1 min. The fruit was then air-dried at ambient temperature and subjected to 3 different treatments, including 400 nL L^−1^ of 1-MCP for 16 h (1-MCP 16 h), 400 nL L^−1^ of 1-MCP for 2 h (1-MCP 2 h), and 0 nL L^−1^ of 1-MCP (control group, CK). All the fruit were then treated with 1000 μL L^−1^ ethephon solution for 1 min, air-dried, and packed in unsealed plastic bags (0.02 mm thick) for ripening at 25 °C. For the control treatment, samples were taken at 0, 1, 2, 4, 6, 8, and 10 days after treatment (DAT). For 1-MCP treatments, samples were taken at 0, 1, 2, 4, 6, 8, 10, and 14 DAT. Samples were immediately snap-frozen with liquid nitrogen and stored at –80 °C for further use for the metabolomic and transcriptomic analyses. Three biological replicates were conducted for all of the treatments.

### 4.2. Transcriptome Sequencing

Nine libraries based on samples that represented the 3 treated fruit from the 6th day of storage were constructed for transcriptome sequencing, as described in our previous work [16]. The samples were designated CK, 1-MCP 2 h, and 1-MCP 16 h. The transcriptome data analysis was conducted as described in our previous work [16].

### 4.3. Sample Preparation and Metabolite Detection

The same 9 samples were used for the metabolic sequencing analysis. The sample preparation, extraction, metabolite identification, and quantification were conducted at Wuhan MetWare Biotechnology Co., Ltd., Wuhan, China (www.metware.cn), as previously described by Guo et al. [71].

### 4.4. Metabolite Data Analysis

Unsupervised principal component analysis (PCA) was conducted using the statistics function prcomp within R (www.r-project.org) [72]. The data was unit variance scaled before unsupervised PCA.

The HCA (hierarchical cluster analysis) results of samples and metabolites were presented as heatmaps with dendrograms. Both the HCA and Pearson correlation coefficients (PCC) were conducted using an R package heatmap. Significantly regulated metabolites between different groups were determined by absolute Log2FC (fold change) ≥ 1 and variable importance in project (VIP) ≥ 1. The data were log-transformed (log2) and mean-centered before OPLS-DA. A permutation test (200 permutations) was performed to avoid overfitting.

The identified metabolites were annotated using the Kyoto Encyclopedia of Genes and Genomes (KEGG) compound database (http://www.kegg.jp/kegg/compound/). The annotated metabolites were then mapped to the KEGG Pathway database (http://www.kegg.jp/kegg/pathway.html) [73]. Pathways with significantly regulated metabolites that mapped were then fed into metabolite sets enrichment analysis (MSEA), and the significance was determined by the hypergeometric test’s *p*-values.

### 4.5. Integrative Analysis of Metabolome and Transcriptome Datasets

For the integrative analysis of the metabolomic and transcriptomic data, the mean of all biological replicates of expression value of differential transcripts in the transcriptome data and differential metabolites in the metabolome data were calculated. Next, the log2 transformed datasets were constructed and loaded in the ‘cor’ package of the R. The Pearson correlation (r) between the transcripts and metabolites was presented by network diagrams, and the differential metabolites and genes between CK, 1-MCP 2 h, and 1-MCP 16 h were selected with R2 >0.8. Transcriptome and metabolome relationships were visualized and presented using the Cytoscape software version 3.6.1 (https://cytoscape.org/).

## 5. Conclusions

The proper treatment with 1-MCP effectively delays the ripening and softening of papaya fruit without causing any adverse effects on fruit quality. However, long-term treatment with 1-MCP causes a ripening disorder of papaya fruit. In this study, the integrated analysis of RNA-Seq and metabolomic data showed that the long-term treatment with 1-MCP caused the ripening disorder that may be closely related to the reduced metabolites, which are primarily flavonoids, lipids, phenolic acids, alkaloids, and organic acids. Energy metabolism pathways, such as the TCA cycle, and downstream secondary metabolic pathways, were significantly inhibited by long-term treatment with 1-MCP. Among the secondary metabolic pathways, the phenylpropane metabolism pathway was dramatically affected by long-term treatment with 1-MCP. The changes in these pathways may account for the physiological disorder caused by 1-MCP. The elucidation of these key molecular factors that regulate fruit ripening and softening may provide useful information for exploring new postharvest storage strategies to maintain and improve fruit quality.

## Supplementary Materials

The following are available online at https://www.mdpi.com/1422-0067/22/2/916/s1.
