# Supplementary figures and images for: Metabolomic and Transcriptomic Profiling Provide Novel Insights into Fruit Ripening and Ripening Disorder Caused by 1-MCP Treatments in Papaya
Source: Int J Mol Sci. 2021 Jan 18;22(2):916. doi: 10.3390/ijms22020916 (PMC7831311; doi:10.3390/ijms22020916)

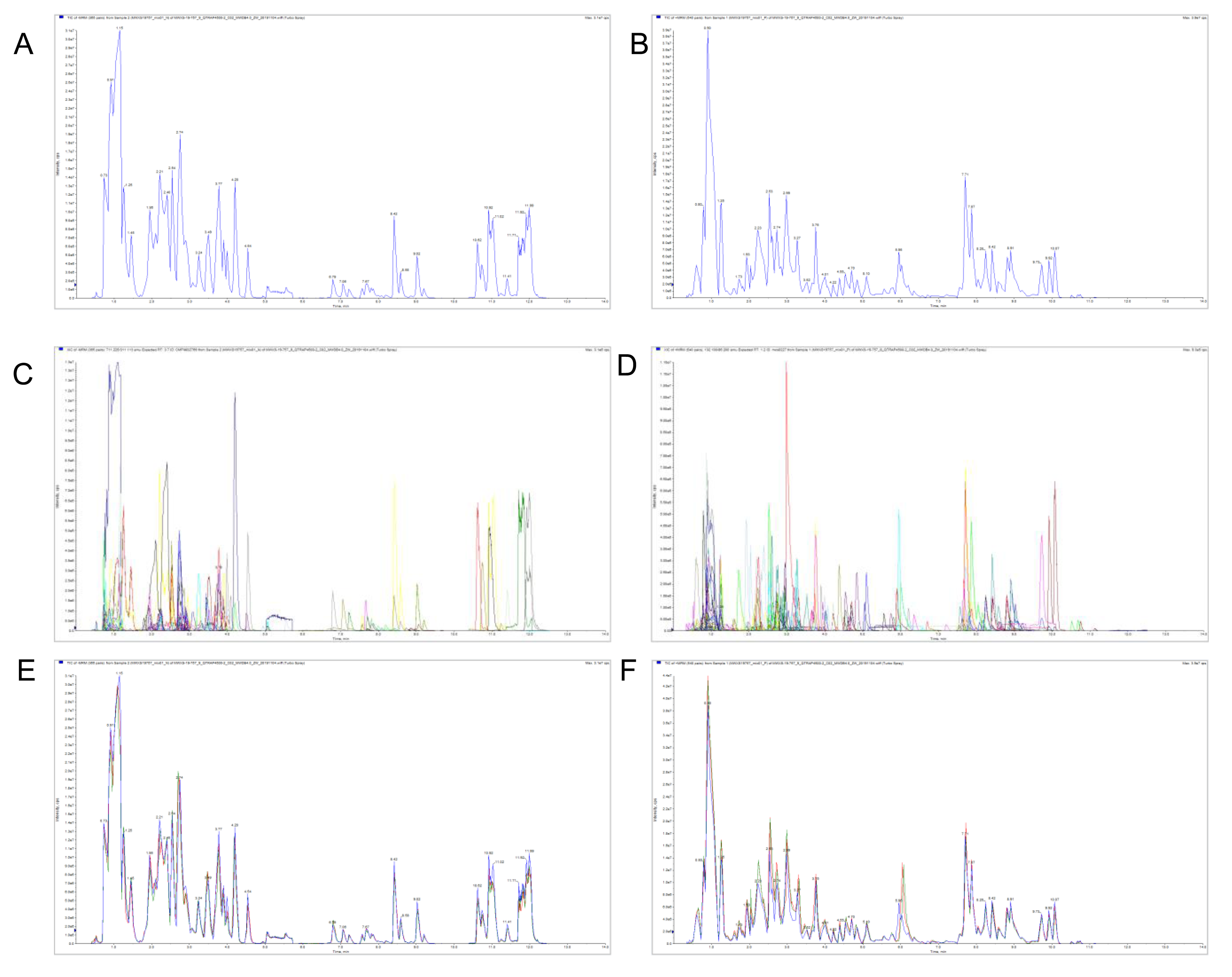

Supplement: Supplementary file 1 [file ijms-22-00916-s001.zip › Supplementary Materials/Figure S1.tif]

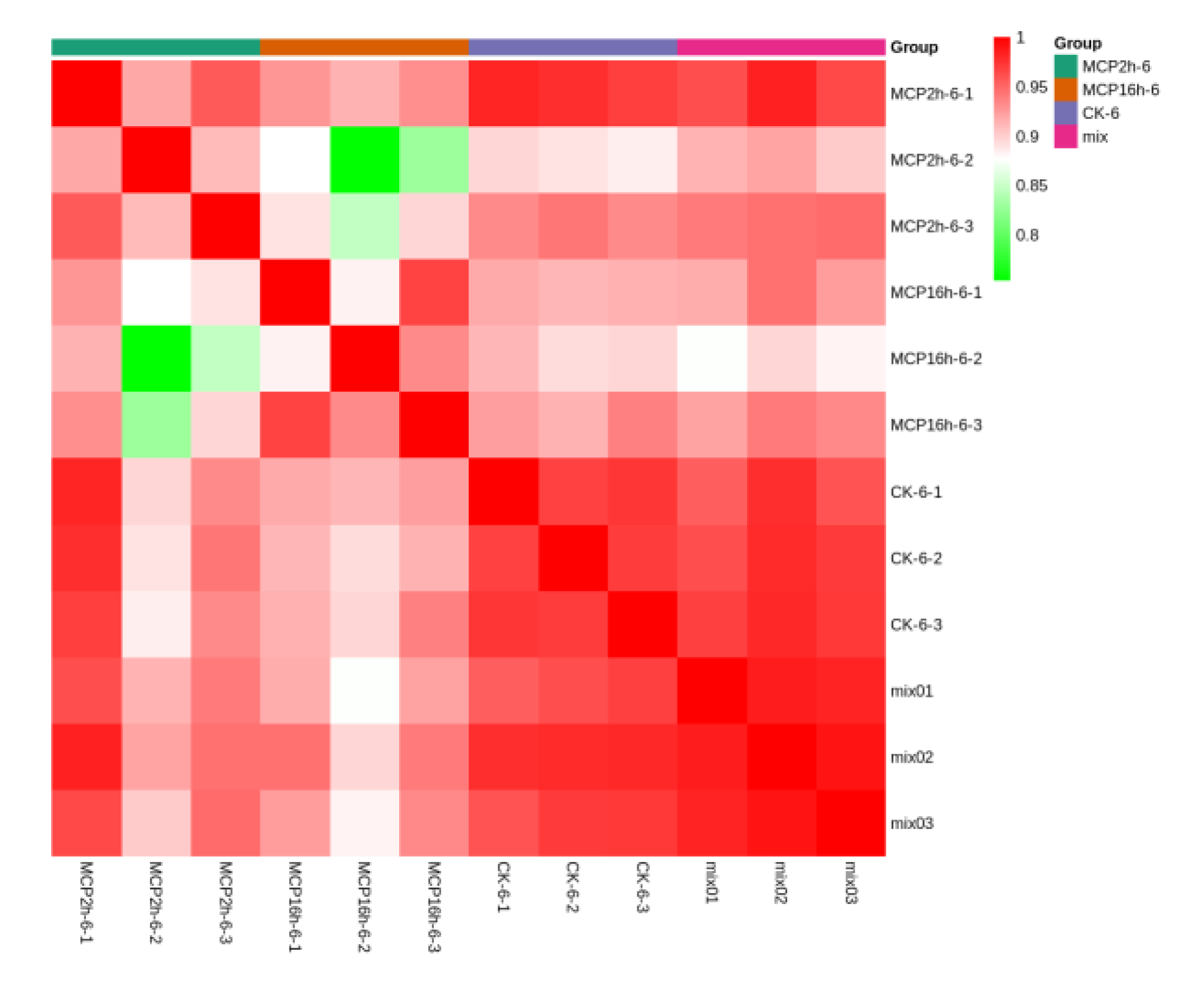

Supplement: Supplementary file 1 [file ijms-22-00916-s001.zip › Supplementary Materials/Figure S2.tif]

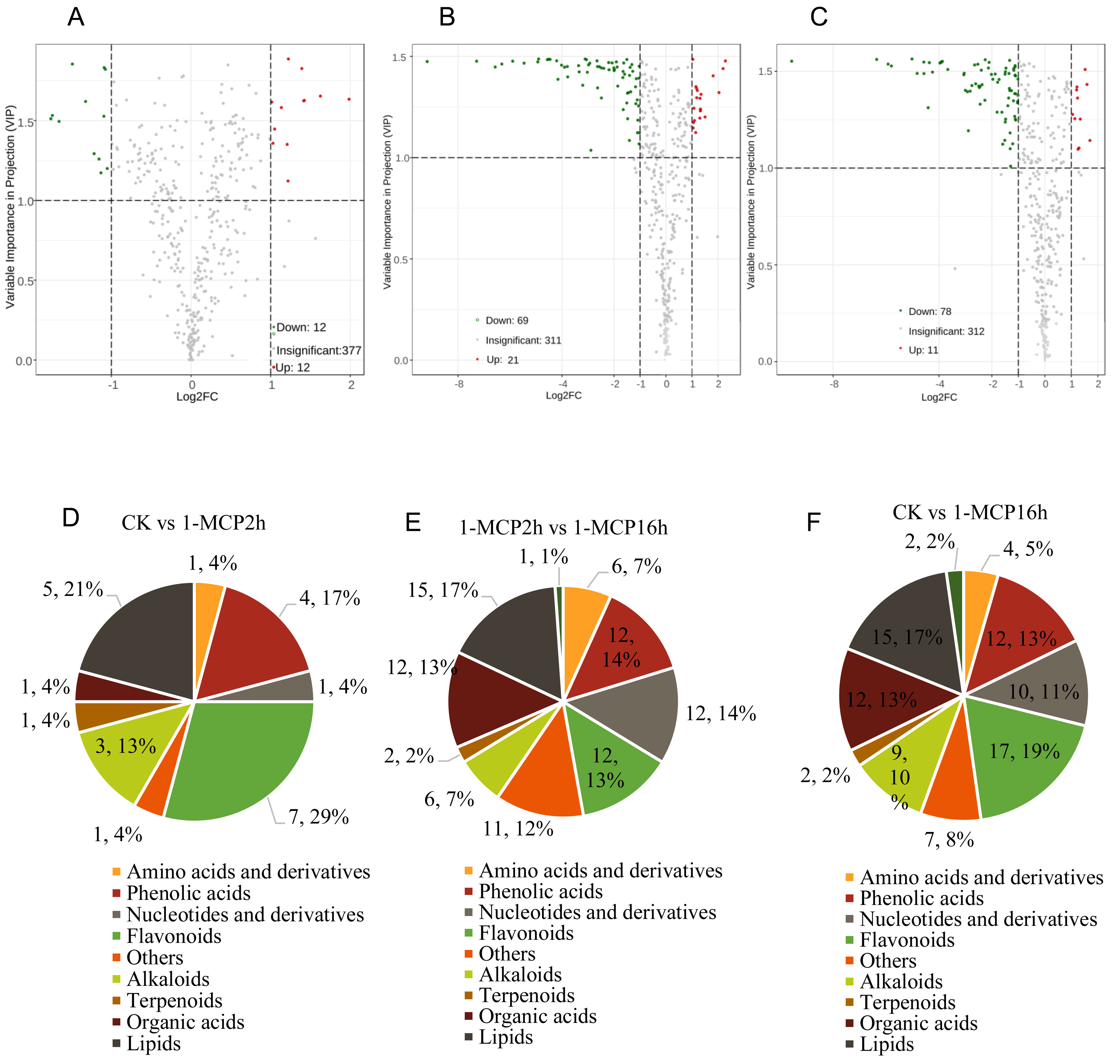

Supplement: Supplementary file 1 [file ijms-22-00916-s001.zip › Supplementary Materials/Figure S3.tif]

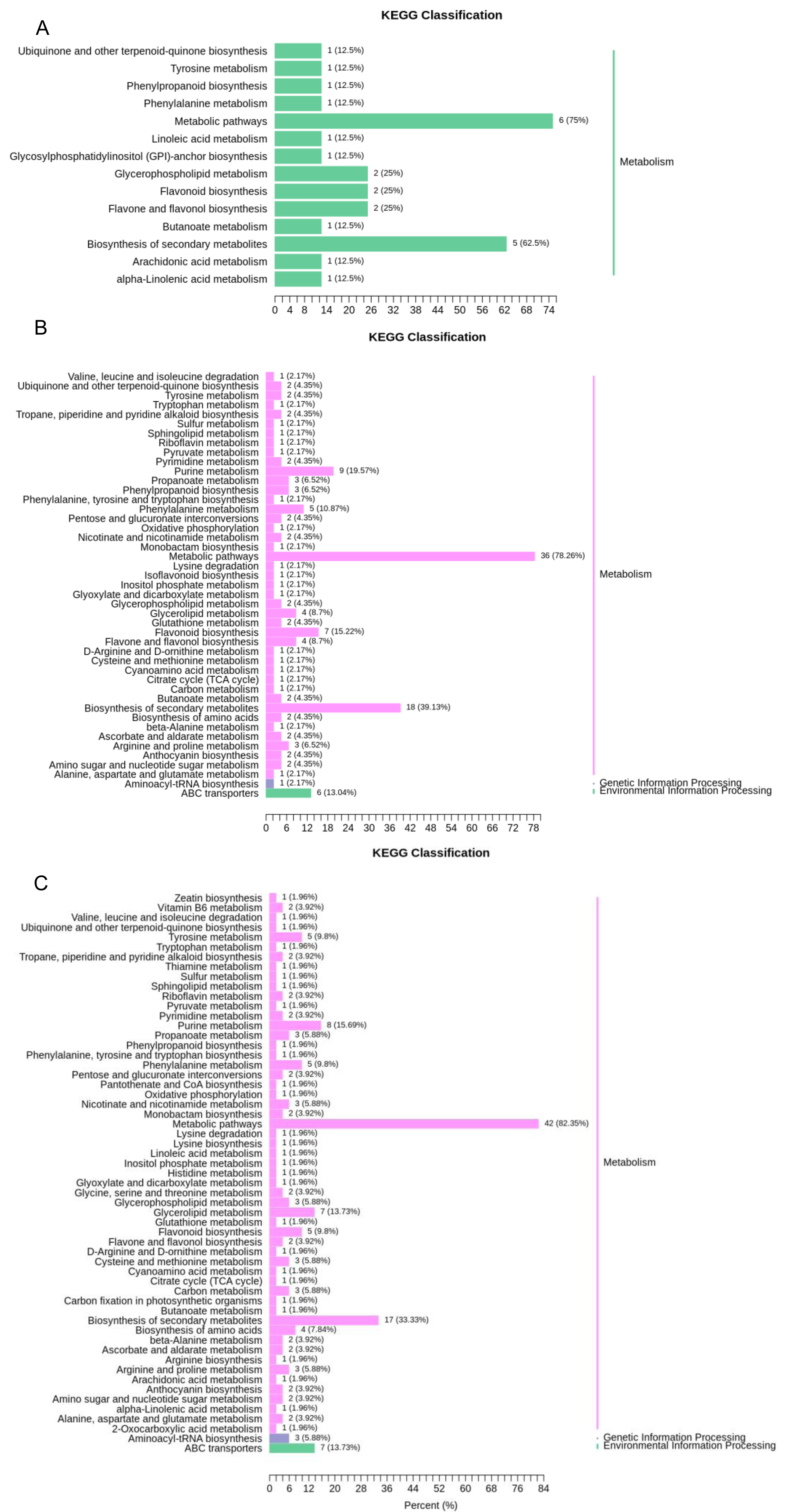

Supplement: Supplementary file 1 [file ijms-22-00916-s001.zip › Supplementary Materials/Figure S4.tif]

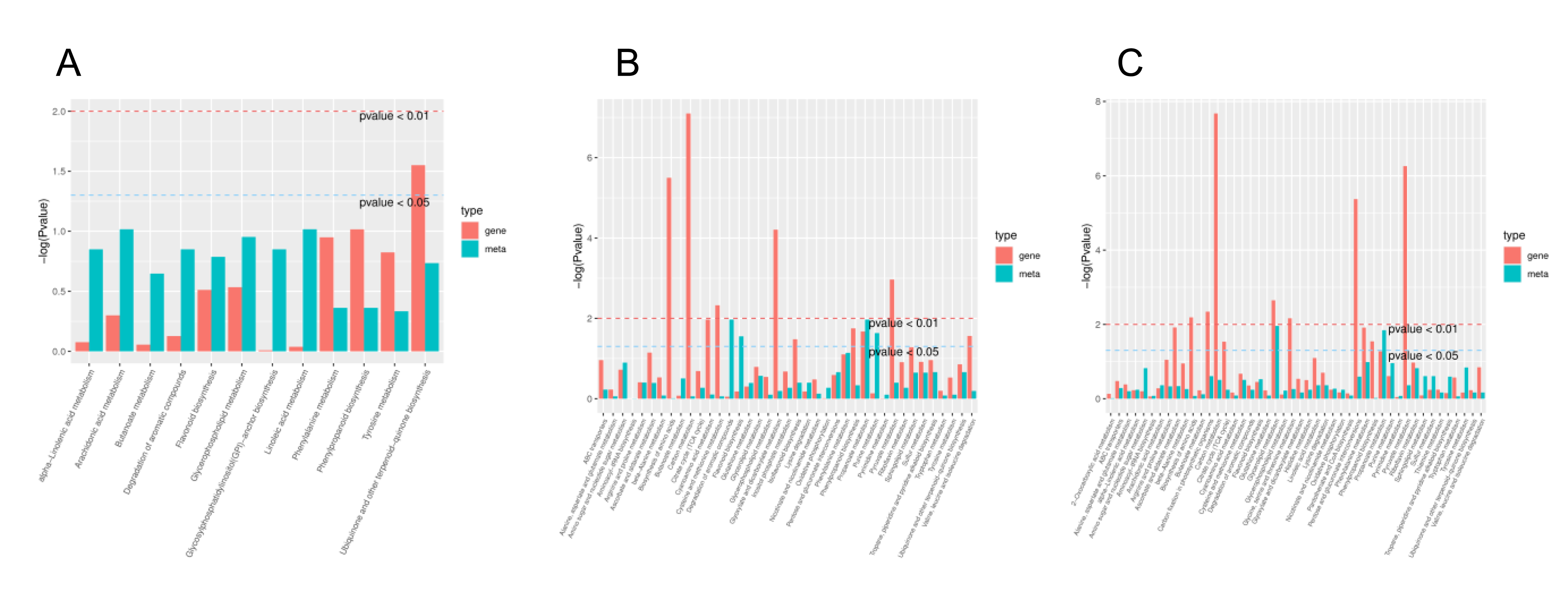

Supplement: Supplementary file 1 [file ijms-22-00916-s001.zip › Supplementary Materials/Figure S5.tif]
